# The prevalence and risk factors of drug allergy in Sri Lanka—a multi-centre cross-sectional observational study (2024–2025)

**DOI:** 10.1016/j.lansea.2026.100804

**Published:** 2026-06-16

**Authors:** Guwani Liyanage, Nadisha Badanasinghe, Dhanushka Dasanayake, Shamini Prathapan, Robert M. West, Wasana Kudagammana, Vaithehi Francis, Nayani Suaraweera, Paula Avello, Pushpika Jayawardana, Mamidipudi Thirumala Krishna

**Affiliations:** a Department of Paediatrics, Faculty of Medical Sciences, University of Sri Jayewardenepura, Sri Lanka; b Department of Medical Microbiology, Faculty of Medicine, University of Kelaniya, Sri Lanka; c Department of Immunology, Medical Research Institute, Colombo, Sri Lanka; d Department of Community Medicine, Faculty of Medical Sciences, University of Sri Jayewardenepura, Sri Lanka; e School of Medicine, University of Leeds, UK; f Department of Microbiology, Faculty of Medicine, University of Peradeniya, Sri Lanka; g Department of Pathophysiology, Faculty of Healthcare Sciences, Eastern University, Sri Lanka; h Department of Paediatrics, Faculty of Medicine, University of Rajarata, Sri Lanka; i Leeds Institute of Health Sciences, University of Leeds, UK; j Department of Paediatrics, Faculty of Medicine, University of Ruhuna, Sri Lanka; k Department of Immunology and Immunotherapy, School of Infection, Inflammation and Immunology, University of Birmingham and Department of Allergy and Immunology, University Hospitals Birmingham NHS Foundation Trust, UK

**Keywords:** Drug allergy, Hypersensitivity, Penicillin, Antibiotics, Sri Lanka, Prevalence

## Abstract

**Background:**

There is a huge burden of drug allergy (DA) in High-Income Countries and is a major impediment to clinical management, adversely affecting outcomes. Data regarding DA from low-and middle-income countries (LMICs) are sparse. Also, there is a vast unmet demand for DA services in LMICs. We aimed to determine the prevalence of self-reported and physician-documented DAs in Sri Lanka and to explore potential risk factors associated with DA labels.

**Methods:**

Cross-sectional, observational study was carried out among adults presenting to outpatient clinics in selected secondary and tertiary care centres during December 2024 to April 2025. A standardised e-proforma in a 1-1 interview captured details. Potential risk factors were explored using multivariable logistic regression.

**Findings:**

Total number of respondents was 12,491: Females: 8410 (67.4%) and males: 4081 (32.6%). Mean age (SD) was 55.1 (14.4) years and 727 reported allergies to 868 drugs; prevalence = 5.8% (95% CI: 5.42–6.24); allergy to ≥2 drugs = 0.74%. Most common reported were antibiotics (41.5%; penicillins most common), nonsteroidal-anti-inflammatory drugs (13%), and paracetamol (10.6%). In the mixed-effect logistic model, odds of DA were lower among males (AOR: 0.74, 95% CI: 0.61–0.88; p < 0.001), and Tamil ethnicity (AOR: 0.67; 95% CI: 0.51–0.89; p = 0.005). Higher odds were observed in 50–<60-year (AOR: 1.55; 95% CI: 1.20–1.99.; p < 0.001) and 60–93-year (AOR: 1.38; 95% CI: 1.08–1.77, p = 0.011) age groups and allergic, endocrine, cardiovascular, rheumatological, neurological, and haematological comorbidities.

**Interpretation:**

There is a high burden of reported DA in Sri Lanka. This is compounded by an unmet demand for DA services highlighting a need for capacity building.

**Funding:**

Institute of Global Innovation, University of Birmingham, UK.

**Research in context**

**Evidence before this study**

A PubMed search was conducted for the period 01 January 2005 to 31 October 2025 using the following search strategies: (a) ‘Drug Allergy’ AND ‘Prevalence’ AND ‘LMIC’ or ‘Low and Middle Income Country’ or ‘Sri Lanka’ or ‘India’ or ‘Pakistan’ or ‘Nepal’ or ‘Bangladesh’ or ‘Burma’; (b) ‘Penicillin Allergy’ AND ‘Prevalence’ AND ‘LMIC’ or ‘Low and Middle Income Country’ or ‘Sri Lanka’ or ‘India’ or ‘Pakistan’ or ‘Nepal’ or ‘Bangladesh’ or ‘Burma’; (c) ‘Antibiotic Allergy’ AND ‘Prevalence’ AND ‘LMIC’ or ‘Low and Middle Income Country’ or ‘Sri Lanka’ or ‘India’ or ‘Pakistan’ or ‘Nepal’ or ‘Bangladesh’ or ‘Burma’. We also reviewed previously published systematic reviews that examined the prevalence of drug and antibiotic allergies. To date, there are no published multi-centre studies from the Indian subcontinent that report on the prevalence or associated risk factors of drug allergy. The vast majority of existing evidence in this field is derived from studies conducted in High-Income Countries.

Drug allergy is a major barrier to effective clinical management and is associated with a range of adverse clinical outcomes. Most drug allergy labels remain unverified, largely because reliable point-of-care diagnostic tests are lacking and formal assessment typically requires evaluation by an allergy specialist. Penicillins are the drugs most frequently implicated in reported allergies, and there is a substantial burden of unverified penicillin allergy labels, estimated at 6–10%, in high-income countries such as the United Kingdom and the United States. Importantly, a penicillin allergy label is itself a risk factor for antimicrobial resistance and is associated with increased rates of hospital-acquired infections, due to the use of broader-spectrum and less optimal antibiotic alternatives.

**Added value of this study**

This study represents the a large, multi-centre investigation from Sri Lanka to report the prevalence of drug allergy in secondary and tertiary care, the drug classes most frequently implicated, and potential risk factors. Our findings indicate that the overall burden of drug allergy in Sri Lanka is lower than that reported in high-income countries such as the United States and the United Kingdom. However, these findings must be interpreted in the context of the substantial unmet demand for drug allergy services, the absence of standardized drug allergy pathways, lack of access to penicillin allergy skin test reagents and the high levels of antimicrobial resistance nationally.

We found that penicillins, non-steroidal anti-inflammatory drugs and paracetamol were the most commonly implicated drug classes in reported allergy. A relatively high proportion of antibiotic allergies were stratified as high-risk. Seven percent of patients with an antibiotic allergy reported self-medication with antibiotics without a valid prescription. Female sex, middle age, urban residence, and the presence of comorbidities, including allergic, cardiovascular, neurological, haematological, and rheumatological conditions were potential risk factors associated with a higher likelihood of reporting a drug allergy.

**Implications of all the available evidence**

This study is an impetus to develop drug allergy services, referral pathways and national guidelines in Sri Lanka. It might need a multi-pronged and a staged approach involving allocation of additional resources, capacity building, investigating facilitators and barriers and adopting a targeted approach by prioritising middle aged patients, those with comorbid condition/s and a penicillin allergy label for drug allergy evaluation in the interest of tackling the global public health threat of antimicrobial resistance.

## Introduction

Drug allergy (DA) labels are a major impediment to effective clinical management, leading to poor clinical outcomes.^1^, ^2^, ^3^ They are usually unverified, and a vast majority are inaccurate.^4^^,^^5^ Broadly, there are two types of adverse reactions to drugs, types A and B.^6^ Type A reactions are common, usually related to the pharmacological property of the drug; they are dose-dependent and reversible upon reducing the dose or withdrawal of the drug. Type B adverse reactions, on the other hand, are relatively rare, unrelated to pharmacological property, and are genetically and/or immune-mediated (drug allergies or hypersensitivity reactions) and dose independent.^6^ Patients with type A adverse drug reactions are commonly mislabelled as ‘drug allergy’ by healthcare professionals, or they may self-report these reactions as an allergy. Published evidence regarding the prevalence of self-reported drug allergies comes mainly from high income countries (HICs) in North America, Europe, and Southeast Asia, with a wide range of 0.7–38.5%.^7^ This variation has been attributed to multiple factors, including differences in study methodologies, population, selection bias, clinical setting, methods of data extraction (interviews, questionnaires or electronic health records) and prospective *versus* retrospective nature of studies.^7^ In HICs, estimates show that drug allergy accounts for 1–2% of hospital admissions, and 14% of emergency room visits. The UK and USA have reported penicillin allergy labels amongst 6–10% of the general population and 15–20% of inpatients.^1^^,^^2^^,^^5^^,^^8^ However, 90–95% of penicillin allergy labels are inaccurate and are associated with an enhanced risk of antimicrobial resistance, hospital-associated infections and higher estimated healthcare costs.^1^^,^^4^^,^^9^, ^10^, ^11^, ^12^, ^13^, ^14^ Epidemiological data regarding drug allergy are sparse in low- and middle-income countries (LMICs), specifically in the Indian subcontinent.^15^

A major challenge in addressing DAs is that there is no simple and reliable point-of-care test.^15^^,^^16^ Drug allergy assessment usually requires a specialist. It is onerous and involves clinical history taking, review of clinical and prescription records, allergy skin tests and, where deemed safe, drug provocative tests.^16^ There is a huge global unmet need for allergy specialists, particularly in LMICs.^16^, ^17^, ^18^, ^19^, ^20^, ^21^, ^22^

Published data on DA prevalence does not exist in Sri Lanka. In a qualitative study, Alqahtani et al.^23^ reported a lack of standardised policies and clear guidelines for managing DAs in Sri Lanka, often leaving healthcare providers uncertain about differentiating true DAs from other adverse drug reactions. Inaccurate penicillin allergy labels were identified as a significant challenge, partly due to easy access to antibiotics without valid prescriptions and the lack of systematic evaluation of reported allergies. Although patients with severe or multiple allergies may be referred to specialists, such services remain scarce due to the limited number of trained allergy specialists and inadequate resources, resulting in unequal access to appropriate management.^20^ Furthermore, penicillin allergy testing is further limited by the lack of diagnostic kits approved by national drug regulatory authorities.

Establishing the prevalence and exploring potential risk factors or correlates of self-reported and physician-documented drug allergy (DA) in Sri Lanka is therefore essential to address this knowledge gap and guide the development of improved services and national policy. Against this background, the main objective of this large, multi-centre cross-sectional study was to determine the prevalence of self-reported and physician-documented DAs in Sri Lanka (referred to as reported DA in this manuscript) and to explore associations between potential risk factors and reported DA. The study was conducted across six secondary and tertiary care institutions covering a wide geographical area and included participants from urban, rural, and estate sectors, thereby ensuring a nationally representative sample.

## Methods

This was a multi-centre cross-sectional observational study conducted in six public sector hospitals across a wide geographical area representing the north, south, east, west, and central provinces of Sri Lanka. The selected centres were Colombo South Teaching Hospital (CSTH; Colombo District), Colombo North Teaching Hospital (CNTH; Gampaha District), National Hospital, Galle (NHG; Galle District), Teaching Hospital, Batticaloa (THB; Batticaloa District), Teaching Hospital, Anuradhapura (THA; Anuradhapura District), and District General Hospital, Nawalapitiya (DGHN; Kandy District).

Unlike in high-income countries, public sector primary curative care services in Sri Lanka are not well developed. As a result, secondary and tertiary care public hospitals function as the first point of contact for many patients, where they are triaged at outpatient departments and either managed or referred to specialist services depending on clinical needs.

The study sites were purposefully selected to represent Sri Lanka’s urban, rural, and estate populations. CSTH and CNTH primarily serve urban communities, DGHN serves estate and rural populations, and NHG, THB, and THA serve mixed populations. Collectively, these sites provide a broadly representative sample. Exclusion criteria included patients unable to provide informed consent, too ill to participate, with significant communication barriers, or previously enrolled in the study.

The study was conducted between December 2024 to April 2025. Adults (≥18 years) attending general triage outpatient units were consecutively invited to participate until the target sample size was reached. Eligible patients received a patient information leaflet in English, Sinhala, or Tamil. Trained research assistants (medical graduates) conducted interviewer-administered questionnaires using a pre-tested e-proforma (Supplementary Table S1) under supervision of the site principal investigator.

A pre-study workshop was held to standardise study procedures. Research assistants were trained via online distance-learning sessions and included detailed guides along with practical exercises. Interviews were conducted in a language preferred by the patient (Sinhala, Tamil or English). Each participant was assigned a unique study identification number.

Demographics including age, gender, ethnicity, literacy, socio-economic status, and comorbid conditions were captured. Respondents were asked: “*Are you allergic to any medicines taken in the past?* (Yes/No)” (as per Sri Lankan standard clinical practice). If “Yes,” details were collected on the names of the drugs, the medical conditions for which they were prescribed, and whether multiple drugs had been taken simultaneously or at separate times. Reported DA was defined as a ‘drug allergy’ documented by the treating physician at the time of the reaction, as well as any allergic reaction to a drug self-reported by the respondent. This included both prescription and non-prescription medications, while vaccines, dietary supplements, and complementary or alternative therapies were excluded. Drug allergy testing was not undertaken in this study.

Participants hand-held clinical record and allergy card (Sri Lanka public healthcare system does not have electronic health records) carried by the respondent were scrutinised and relevant data were captured. Additional relevant information was also collated from a review of all medical records. If a participant did not have their clinical record and/or allergy card to hand and was unable to provide adequate details of their allergy status at the time of the interview, information was collected later via telephone (with prior consent). If documentation was available and could be reviewed, the allergy was classified as physician documented. If no documentation was available, the allergy was classified as self-reported. If the respondent was unable to provide the name of the drug, or if the drug could not be identified after reviewing available records, the reaction was categorised as due to an ‘unknown drug’ or ‘unknown antibiotic’.

Patients reporting ‘allergic reactions to antibiotics’ underwent a more detailed interview, and their hand-held clinical records were reviewed to obtain information on the name of the implicated antibiotic and the nature of the reaction. If allergy was reported to multiple antibiotics, this information was obtained for each antibiotic. Patients were stratified as ‘low-risk’ and ‘high-risk’ based on published criteria.^4^^,^^24^ Risk stratification outcome for each participant was reviewed and ratified by a panel of senior clinicians with an expertise in allergy during weekly online meetings during the study period. During these online meetings, all data were reviewed by the investigators to mitigate recording errors and maintain uniformity and standardisation between participating centres. Hypersensitivity reactions were classified as immediate or delayed based on timing of onset, with immediate reactions defined as occurring within 6 h of drug exposure and delayed reactions as those occurring after the second dose or later during or after completion of therapy. Where participants were unable to recall the timing of the reaction, events were classified as indeterminate.

Data were collected in real time using EpiCollect5 (Centre for Genomic Pathogen Surveillance. 2025. Epicollect5. Available at: https://five.epicollect.net), a mobile application developed for field data collection. All data collectors used the same application, and the data were subsequently exported to Microsoft Excel and IBM SPSS (version 25) for cleaning and analysis.

### Statistical analysis

The sample size calculation was based on a prior clinical audit conducted in Colombo, Sri Lanka (Liyanage G et al., 2023, unpublished), which reported self-reported and physician-documented DA in 12% of patients in secondary care, with antibiotics, NSAIDs, and radiocontrast media being the most commonly implicated drugs. Based on this, a sample size of 9600 was calculated to estimate a 12% prevalence with a ±0.65% margin of error at 95% CI. Proportionate sampling distributed the sample across districts according to adult population demographics (Census and Population Estimates, 2023), enhancing representativeness and precision. During recruitment, the total sample size was increased by approximately 30% (12,480 rounded up to 12,500) for two reasons. First, proportionate sampling yielded small numbers from four districts (Kandy, Galle, Batticaloa, and Anuradhapura). Second, the observed prevalence of DA in these districts was lower than anticipated, risking insufficient numbers for reliable subgroup analyses. This increase preserved statistical power, accommodated potential missing data, and ensured accurate representation across districts.

Descriptive statistics were computed to assess the prevalence of DA across the sample. Frequencies and percentages were reported for categorical variables. The prevalence of DA with 95% confidence intervals (CIs) was calculated for the overall sample, as well as stratified by age and sex. Bivariate associations between allergy to any drug (dependent variable) and independent variables were evaluated using Pearson’s chi-square tests. Crude odds ratios (ORs) and 95% CIs were calculated to quantify the strength of associations. A p-value of <0.1 was considered statistically significant for an exploratory analysis. A mixed-effects logistic regression model was constructed with an allergy to any drug as the dependent variable. Predictor variables including demographic and health-related characteristics were incorporated as fixed effects. A random intercept for Centre was included to account for within-centre correlation. Variables for the multivariable regression model were selected based on a combination of theoretical relevance, prior literature, and univariable analysis. Variables that were statistically significant in the univariate analysis and that showed at least a modest effect size, were subsequently included in the multivariable model. A threshold of OR ≥ 1.2 was applied as a modest effect size. Adjusted ORs and 95% CIs were reported to determine the strength and direction of associations while controlling for potential confounders. Model assumptions, including multicollinearity, were tested prior to regression. Variance inflation factor (VIF) values < 5 indicate acceptable levels of collinearity. The intra-class correlation coefficient (ICC) and the random-intercept variance were calculated to quantify between-centre variability and to support the use of a mixed-effects logistic regression model.

### Ethics statement

Ethical approval for this study, including the amendment to the sample size, was obtained from the Ethics Review Committee of the Faculty of Medicine, University of Peradeniya (2024/EC/08). The study adhered to the principles of the Declaration of Helsinki. Written informed consent was obtained from patients/carers prior to participation.

### Role of the funding source

The funding source had no role in the study design, data collection, analysis, interpretation, or writing of the manuscript.

## Results

**Fig. 1:**
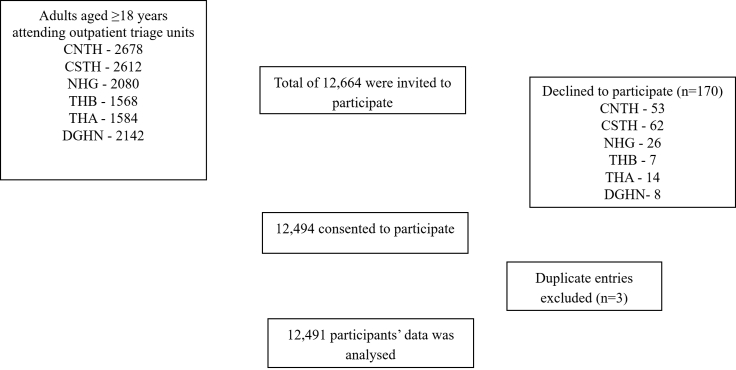
Flow chart for recruitment.

A total of 12,664 were invited to participate. One hundred and seventy declined. Three duplicate entries were removed. A complete dataset was available for 12,491 patients. The study flow diagram is shown in Fig. 1. The mean (±SD) age of the participants was 55.1 (14.4) years. The female-to-male ratio was 544 (74.8%) to 183 (25.2%), corresponding to a ratio of 2.97 (Supplementary Table S2). Among them, 727 individuals (5.8%; 95% CI: 5.42%–6.24%) reported DA. Of these, approximately one-third (n = 279, 38.4%) reported an allergy to antibiotics. The majority reported allergy to one drug (635/727, 87%), while 92/727 (12.6%) to two or more drugs (Table 1). Thus, it accounted for 868 reported DA [6.9% (95% CI: 6.5%–7.4%)].

**Table 1:** Frequency distribution of single and multiple reported drug allergies in participants.

Number of drug allergies	N = patients	Percentage
1	635	87.3
2	59	8.1
3	21	2.9
4	9	1.2
5	2	0.3
6	1	0.1
Total	727	

As age increased, the proportion of participants reporting a DA also increased, with 70.3% of all reported DA occurring among individuals over 50 years of age, and the highest representation in the 60–93 age group (43.1%). This pattern was observed in both males and females, although females are more represented overall. These data are summarised in Supplementary Table S2. The highest rate of DA was reported from Colombo and Gampaha and crosstabulation of patient characteristics by study site demonstrated notable variation in case mix across districts (Supplementary Tables S3 and S4). Colombo and Gampaha which are predominantly urban, had a higher proportion of older participants. Other districts had lower rates of comorbid conditions such as allergic disorders, hypertension and metabolic disorders compared to Colombo and Gampaha.

The leading drug classes implicated were antibiotics (N = 360/868; 41.5%), NSAIDs (N = 113/868; 13%) and paracetamol (N = 92/868; 10.6%), followed by other drug classes. Among antibiotics, penicillins accounted for the majority (65%) of reported allergies. These data are summarised in Table 2. Fifty-two participants (6%) reported an allergy to an unknown antibiotic, 68 (7.8%) to an unknown analgesic, and 82 (9.4%) to an unknown drug.

**Table 2:** Frequency distribution of the drugs and its class.

Drug class	Drug	N=	%
Antibiotics			
	**Penicillins**		
	Amoxicillin	99	11.4
	Penicillin	68	7.8
	Ampicillin	28	3.2
	Co-amoxiclav	24	2.7
	Cloxacillin	15	1.7
	**Fluoroquinolones** (Ciprofloxacin)	20	2.3
	**Cephalosporins** (cephalexin, ceftriaxone, cefuroxime)	18	2.1
	**Sulphonamides**	12	1.4
	**Macrolides**	11	1.2
	**Tetracycline**	4	0.5
	**β lactams (Name not specified)**	1	0.1
	**Other antibiotics**^b^		
	Metronidazole	2	0.2
	Furazolidone	1	0.1
	Gentamicin	1	0.1
	Meropenem	1	0.1
	Nalidixic acid	1	0.1
	Nitrofurantoin	1	0.1
	Rifampicin	1	0.1
	Unknown Antibiotic	52	6.0
Nonsteroidal anti-inflammatory drugs	NSAIDs	113	12.7
Paracetamol	Paracetamol	92	10.6
Antiseptics	Betadine	14	1.6
Paracetamol and codeine phosphate	Paracetamol and codeine phosphate	12	1.4
Unknown analgesics	Unknown analgesics	68	7.8
Unknown drugs	Unknown	82	9.4
Other drug classes^a^	Other drug classes^a^	127	14.6

a ACE inhibitors, anaesthetics, alpha blockers, anti-helminthic, antimalarial drugs, anti-rabies vaccine, anti-cancer/tyrosine kinase inhibitor, anticonvulsants, antidiarrheal, anti-emetic, antihistamines, antiplatelet, antiprotozoal, antispasmodic, calcium channel blocker, coagulant factor supplement, disease modifying antirheumatic drug, H2-receptor blocker, histamine analogue, hormone, immunoglobulin, laxative, lipid lowering agent, nitrate, non-allergic plaster, opioid, oral contraceptive pill, oral hypoglycaemic agent, proton pump inhibitor, sclerotherapy agent, sildenafil, steroids, thyroid hormone, unknown anaesthetic, unknown antiepileptic, unknown antirheumatic, vitamins and minerals, beta adrenergic receptor agonist.

b Furazolidine, metronidazole, gentamycin, meropenem, nalidixic acid, nitrofurantoin, rifampicin.

Most reported allergy events were classified as high risk (n = 215, 65.4%) compared to low risk (n = 114, 34.6%). High-risk categorisation predominated across all groups, including beta-lactams (64.5%), non-beta lactams (68.8%), and unknown drugs (68.0%) (Table 3). Among 302 reported antibiotic allergy occurrences, most were due to beta-lactams (n = 239, 79.1%), followed by non-beta lactams (n = 39, 12.9%) and unknown antibiotics (n = 24, 7.9%). Immediate hypersensitivity reactions predominated across all groups: 78.2% in beta-lactams, 76.9% in non-beta lactams, and 91.7% in unknown antibiotics. Delayed reactions were less common in all categories Table 4.

**Table 3:** Risk categorisation for reported allergy events.

	Low Risk (n = 114)	High Risk (n = 215)
Allergy to any beta lactam (%)	91 (35.5)	165 (64.5)
Allergy to any Non beta lactam	15 (31.3)	33 (68.8)
Unknown (%)	8 (32.0)	17 (68.0)

**Table 4:** Type of hypersensitivity reactions to reported antibiotic allergies.

	N = 329 (%) of occurrences	Immediate HSR,^a^ N = 239 (72.6%)	Delayed HSR, N = 63 (19.1%)
Beta lactam antibiotic	262 (79.6)	187 (76.9)	52 (82.5)
Non-beta lactam antibiotic	42 (12.8)	30 (12.5)	9 (14.3)
Unknown antibiotic	25 (7.6)	22 (9.2)	2 (3.2)

Reactions were classified as immediate or delayed based on timing of onset. Where participants were unable to recall the timing of the reaction, events were classified as indeterminate (n = 27) and excluded from analyses comparing immediate and delayed hypersensitivity reactions.

a Some immediate reactions (e.g., mild cutaneous eruptions) were classified as low risk by the expert panel.

Seven percent of all study participants regardless of their allergy status reported self-medication with antibiotics without a valid prescription from a healthcare professional. Reported DA was slightly higher among individuals who self-medicated, showing a trend towards increased odds, although this did not reach statistical significance (OR = 1.46; 95% CI: 0.99–2.17; p = 0.054). Forty percent of patients carried an allergy card listing their DA and 89% reported making their doctor aware regarding their DA prior to receiving treatment. These data are summarised in Table 5.

**Table 5:** Health Care behaviour of the participants related to medication.

Variable	N	Percentage
Self-medication with antibiotics without a valid prescription from a healthcare professional amongst all (n = 12,491) study participants	871	6.97
The number of participants (out of 727) who carried an allergy card when visiting healthcare facilities indicating their drug allergy	293	40.30
Number of participants (out of 727) that would inform healthcare professional about drug allergy status while seeking treatment	647	88.99

**Fig. 2:**
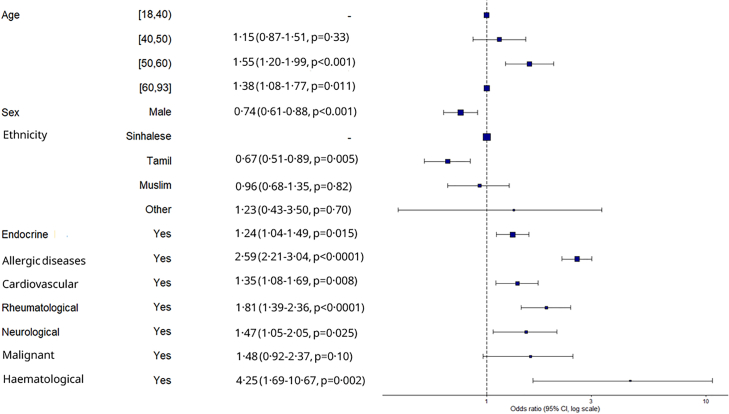
Odds ratio plot showing the effect sizes of selected variables (Boxes represent the odds ratios, and horizontal lines indicate the 95% confidence intervals).

In the multivariable model (Table 6), the odds of having a reported DA were significantly lower among males compared to females (AOR 0.74; 95% CI: 0.61–0.88; p < 0.001). Participants in the 50–<60-year and 60–93-year age groups had statistically significant higher odds of having a reported DA compared to the reference group (18–<40 years). In comparison to participants of Sinhalese ethnicity, those of Tamil ethnicity demonstrated a significantly lower odds of reported DA (AOR 0.67; 95% CI: 0.51–0.89; p = 0.005), while no significant difference was observed with Muslim ethnicity. The odds were also significantly increased in individuals with comorbid conditions. Allergy-related conditions were associated with nearly a threefold increase in the odds of drug allergy labels (AOR 2.59; 95% CI: 2.21–3.04; p < 0.0001). Rheumatological disorders were associated with approximately a twofold increase in the odds (AOR 1.81; 95% CI: 1.39–2.36; p < 0.001). In addition, cardiovascular disorders (AOR 1.35, 95% CI: 1.08–1.69; p = 0.008), neurological disease (AOR 1.47; 5% CI: 1.05–2.05; p = 0.025), endocrine (AOR 1.24; 95% CI: 1.04–1.49; p = 0.015) and haematological disorders (AOR 4.25; 95% CI: 1.69–10.67; p = 0.002) were also associated with significantly higher odds of having a reported DA. Although hypertension, metabolic, and other categories showed significant associations in the univariable analysis, these were not retained after adjustment in the multivariable model. These data are summarised in Table 6 and illustrated in Fig. 2, where the odds-ratio plot highlights the effect sizes and the uncertainty around them.

**Table 6:** Univariable and multivariable logistic regression analysis of potential risk factors associated with reported drug allergy.

	Total N	Yes, N (%)	OR (univariable)	OR (multivariable)
Age				
18–<40	2875	107 (3.7)	–	–
40–<50	2321	109 (4.7)	1.26 (0.96–1.66)	1.15 (0.87–1.51)
50–<60	2789	198 (7.1)	1.88 (1.48–2.41)	1.55 (1.20–1.99)
60–93	4506	313 (6.9)	1.77 (1.41–2.24)	1.38 (1.08–1.77)
Sex				
Female	8410	544 (6.5)	–	–
Male	4081	183 (4.5)	0.64 (0.54–0.76)	0.74 (0.61–0.88)
Ethnicity				
Sinhalese	9486	597 (6.3)	–	–
Tamil	2159	83 (3.8)	0.62 (0.46–0.82)	0.67 (0.51–0.89)
Muslim	795	43 (5.4)	0.90 (0.63–1.26)	0.96 (0.68–1.35)
Other	53	4 (7.8)	1.17 (0.35–2.90)	1.23 (0.43–3.50)
Comorbidities				
Endocrine	3089	248 (8.0)	1.46 (1.24–1.72)	–
Allergic diseases	2770	300 (10.8)	2.67 (2.28–3.13)	–
Cardiovascular	1314	112 (8.5)	1.50 (1.21–1.85)	–
Renal	648	52 (8.0)	1.24 (0.91–1.68)	–
Rheumatological	634	81 (12.8)	2.26 (1.75–2.90)	–
Neurological	476	42 (8.8)	1.42 (1.01–1.95)	–
Malignant	220	21 (9.5)	1.57 (0.96–2.43)	–
Chronic urticaria	220	16 (7.3)	1.22 (0.70–1.98)	–
Chronic liver cell disease	169	11 (6.5)	1.01 (0.51–1.79)	–
Mental illness	148	9 (6.1)	1.03 (0.48–1.92)	–
Respiratory	34	2 (5.9)	1.10 (0.18–3.67)	–
Dermatological	44	3 (6.8)	0.96 (0.23–2.65)	–
Haematological	30	6 (20.0)	3.59 (1.32–8.32)	–
Gastrointestinal	15	2 (13.3)	2.45 (0.38–9.00)	–
Hypertension	3689	288 (7.8)	1.47 (1.26–1.73)	–
Metabolic conditions	3027	249 (8.2)	1.51 (1.28–1.78)	–
Other	397	45 (11.3)	1.84 (1.31–2.52)	–

## Discussion

This is a multi-centre cross-sectional study from Sri Lanka reporting the prevalence of reported DA, the implicated drug classes, and associations with potential risk factors (correlates). The overall prevalence of reported DA was 5.8% and the top three leading drug classes implicated were antibiotics, NSAIDs, and paracetamol. Amongst antibiotics, penicillins were most implicated.

The burden of reported DA in Sri Lanka seems relatively less compared to those reported in HICs^5^^,^^7^^,^^25^ For example, large studies involving interrogation of primary care databases in the UK showed that the prevalence of unverified penicillin allergy labels alone is 6–8%, and estimates suggest even higher rates in the USA and Australia.^1^^,^^2^^,^^4^^,^^26^ In a cross-sectional study involving territory wide anonymized electronic patient data in Hong Kong, the prevalence of unverified beta lactam allergy was 2%.^27^ The prevalence in DA in Sri Lanka (5.8%) is similar to that observed in another large population-based study from Hong Kong (5.6%) involving over 7 million individuals.^28^ The most common drugs implicated were antibiotics, NSAIDs and nervous system drugs.^28^ Among LMICs, Christopher et al. reported a prevalence of reported DA at 5.8% in adults in a specialist pulmonary unit in Vellore, South India, which is similar to the findings of our study.^29^ In LMICs, limited access to healthcare, underreporting, less systematic record-keeping, and lower patient awareness may contribute at least in part to lower prevalence compared to HICs. However, these comparisons should be interpreted with caution, considering potential methodological differences, variations in literacy and socioeconomic status of participants, as well as differences in cultural and religious factors, health service frameworks and clinical settings.

Whilst the burden of reported DA appears relatively lower in Sri Lanka compared to HICs, the drug classes implicated are similar, with penicillins and NSAIDs being the most frequently reported. However, Christopher et al., reported that sulphonamides were more frequently implicated than penicillins in the Vellore study.^29^ Interestingly, paracetamol was the third most commonly implicated drug in our study, with an overall prevalence of 1% in the study cohort. This contrasts with findings from HICs, where anaesthetic agents typically represent the third leading category after penicillins and NSAIDs.^7^ A moderately high proportion of patients reporting an antibiotic allergy were stratified as high-risk in this study (64.5–68.0%).

Interestingly, 7% of respondents reported self-medicating with antibiotics without a valid prescription from a healthcare professional. This is higher than previously reported in the Anuradhapura district^30^ but lower than estimates from Asia (26%).^31^ Such practices may lead to unnecessary antibiotic exposure and potentially increase the risk of sensitisation; further research is needed to better understand this association.

The potential risk factors (correlates) identified in the multivariable mixed-effects logistic regression analysis were age, female sex, Sinhalese ethnicity (compared to Tamil ethnicity), and comorbid conditions. Individuals aged 50 years and above showed a higher risk of reported DA compared to the younger group (18–<40 years). This pattern may reflect differences in health-seeking behaviour and reporting tendencies among middle-aged and older adults, who are generally more engaged with healthcare services and more likely to recall or report previous adverse drug reactions. The reason underpinning the enhanced risk of Sinhalese ethnicity compared to Tamil ethnicity is uncertain and was not within the scope of our work to investigate further. There is very limited literature surrounding ethnicity-based variations and disparities in DA. A recent study from the USA reported an inverse association between social vulnerability and penicillin allergy labels, with significant racial disparities with respect to greater odds of penicillin allergy labels occurring amongst non-Hispanic American White population compared to Hispanics and non-Hispanic Black and non-Hispanic Asian Americans.^32^ In our study, allergic, cardiovascular, rheumatological, haematological, endocrine and neurological conditions showed a statistically significant association with reported DA. Repeated drug exposures, cumulative labelling and polypharmacy may partly explain these associations rather than enhanced biological susceptibility to immunologically mediated hypersensitivity due to underlying comorbidity *per se*. Interestingly, no such associations were seen with chronic urticaria. Jani et al., in the UK study, reported asthma, diabetes, and chronic kidney disease as risk factors for unverified penicillin allergy labels.^2^ Recognizing the significant association of drug allergy labels among individuals with selected comorbid conditions is important, as it allows for targeted interventions in people with comorbidities. To account for clustering across districts, a mixed-effects model was employed with district included as a random effect. The low intraclass correlation coefficient (ICC = 0.007) and minimal between-district variability (SD = 1.16) suggest that variation in reported drug allergy is largely attributable to individual-level factors rather than district-level differences.

This is a large study, involving 12,491 patients. It utilised one-to-one interviews by trained research personnel, employing a standardised e-proforma. Consecutive patients presenting from the community across a wide catchment area, including rural, urban, and estate populations were enrolled through a triage outpatient clinic, thereby minimising potential bias associated with specialist unit-based studies. This study systematically assessed patient demographics, including age, gender, ethnicity, literacy, and socioeconomic status, along with documentation of drug names, characterisation of drug classes, data on comorbidities, and review of patient-held healthcare records. This data allowed mixed-effects logistic regression modelling to explore potential risk factors for reported DA in Sri Lanka. The study had a few limitations. First, it was beyond the scope of this project to conduct DA testing, therefore our data does not report ‘true’ DA prevalence. Secondly, children (<18 years) were not included. Third, patients presenting to fee levying private sector hospitals were not included. The latter two groups may have different patterns of drug exposure and drug allergy labelling. Fourth, our findings are potentially subjected to variability in healthcare access, cultural factors, literacy and DA labelling practices across different settings, and it was not within the scope of this study to disentangle the potential influence of these confounders. Fifth, this dataset does not reflect the burden of reported DA in the community. Due to the cross-sectional nature of the study, causality cannot be inferred. The observed associations should therefore be interpreted as exploratory.

This study highlights a need for establishment of referral pathways and standardised national protocols for DA management in Sri Lanka. An important challenge is that allergy and immunology is not recognised as a standalone specialty in Sri Lanka. The limited allergy services currently available are provided by a small number of organ-based specialists in Colombo with restricted training opportunities and lack of access to penicillin allergy skin test reagents.^18^, ^19^, ^20^, ^21^, ^22^ The latter is of particular relevance in the context of a relatively high proportion of patients with beta lactam allergies stratified as high-risk in this study. A staged approach for improving DA services may be considered, focusing on antibiotics, NSAIDs, and paracetamol first, as they comprise 65% of the reported DAs. Penicillin allergy is a high-priority area, as de-labelling inaccurate allergies will feed into the WHO AWaRe framework and address high rates of AMR, a global public health problem. Secondly, a trained non-allergy healthcare professional led service could provide an opportunity to implement a computerised decision support system to facilitate penicillin allergy de-labelling in ‘low-risk’ patients, as highlighted in a previous qualitative study in Sri Lanka,^23^ as well as an integrative and joined up strategy with local antimicrobial stewardship is worth exploring. Thirdly, despite poor resource availability, identifying at-risk groups such as older adults and individuals with comorbid conditions and implementing targeted interventions might help optimise use of resources. Fourth, further research into an in-depth characterisation including outcome of allergy tests and challenges in low-risk and high-risk patients to determine what proportion are truly allergic might feed into a robust and safe bespoke stratification process of Sri Lankan patients for a direct oral penicillin challenge, resource planning, business case development and policy making.

In conclusion, there is a high burden of unverified DA in Sri Lanka, with antibiotics, NSAIDs, and paracetamol most frequently implicated. A multi-pronged approach, including capacity building, allocation of additional healthcare resources, qualitative research to identify facilitators and barriers, and knowledge transfer from specialist DA centres in HICs is warranted.

## Contributors

GL: study design, data collection, data analysis, data interpretation, writing- original draft, and writing-review & editing. TK: study design, data analysis, data interpretation, writing- original draft, and writing-review & editing. NB, DD, WK: study design, data collection, data interpretation, and writing-review & editing. RMW, SP: study design, data analysis, data interpretation, and writing-review & editing. PA: data analysis, data interpretation, and writing-review & editing. VF, NS, PJ: data collection, data interpretation, and writing-review & editing. All authors had full access to all the data in the study and had final responsibility for the decision to submit for publication. GL, TK, SP, RMW, and PA accessed and verified the data.

## Data sharing statement

De-identified participant data will be made available upon reasonable request from the corresponding author.

## Declaration of interests

TK was a co-author of BSACI guidelines on penicillin allergy and served as chief investigator for NIHR funded SPACE study (NIHR129069). TK also received research grants from NIHR, MRC CiC (via University of Birmingham), GCRF (via University of Birmingham) and FSA UK for research outside the work presented in this manuscript. All the other authors declare no competing interests.
