# Supplementary material for: The prevalence and risk factors of drug allergy in Sri Lanka—a multi-centre cross-sectional observational study (2024–2025)
Source: Lancet Reg Health Southeast Asia. 2026 Jun 16;51:100804. doi: 10.1016/j.lansea.2026.100804 (PMC13285830; doi:10.1016/j.lansea.2026.100804)
Supplement: Supplementary Tables S1–S5 [file mmc1.docx]

**Table S1: Electronic questionnaire employed in the study for data collection**

**The Burden of Penicillin and other Antibiotic Allergy Labels and Antimicrobial Resistance in Sri Lankan Adults**

**Aims and objectives of work package 1:**

This study is done to determine the prevalence of unverified drug allergy labels (DALs), prevalence of unverified antibiotic allergy labels (AALs) (including penicillin allergy labels-PALs) and multiple AALs in SL, to determine what classes of antibiotics are commonly implicated in an allergy in SL, what are the clinical characteristics of patients with AALs in SL and to risk stratify AALs in SL.

**Instructions to interviewers**

The questionnaire is divided to 3 parts. You will be assigned an enumerator code.

Part A- Demographic data and comorbidities

Part B- Information on drug allergy

Part C- Information on antibiotic allergy

Some questions have only one answer to be marked whereas some questions require multiple answers to be marked.

**Part A- DEMOGRAPHIC DATA AND COMORBIDITIES**

A1: Start Time- Automatic

A2: Date of interview- Calendar with the date marked

A3: Center name – (drop down to select one option)

CSTH

CNTH

Karapitiya

Anuradhapura

Batticaloa

Nawalapitiya

A4: Enumerator code – (drop down to select one option)

CSTH1

CSTH2

CNTH1

CNTH2

K1

K2

A1

A2

B1

B2

NW1

NW2

A5: Patient study number - type

A6: Clinical setting – (drop down to select one option)

General OPD

Specialist Outpatient clinic

A7: Age – type

A8: Gender – (drop down to select one option)

Male

Female

A9: Ethnicity- (drop down to select one option)

Sinhalese

Tamil

Muslim

Other

A10: Level of education- (drop down to select one option)

No formal education

Up to primary school

Up to secondary school (completed OL)

Up to secondary school (not completed OL)

Up to high school (completed AL)

Up to high school (not completed AL)

Higher (diploma, degree, etc.,)

A11: Socio-economic status/ Monthly household income (LKR) – (drop down to select one option)

<50,000

50,000-100,000

100,000-200,000

` >200,000

A12: Chronic illness – Select one/ more option – (drop down to select multiple options)

Allergic conditions (asthma, rhino-conjunctivitis, food allergy)

Chronic urticaria

Chronic liver diseases

Renal disease

Cardiovascular disease

Respiratory conditions (other than asthma)

Hypertension

Malignancy

Metabolic conditions

Endocrine problems

Neurological conditions

Rheumatological conditions

Mental health illness

Any other – If this is selected, 3 spaces are be provided

A13: Do you take antibiotics over the counter or as self-prescribed? (drop down to select one option)

Yes

No

A14: Are you allergic to any drugs? - (drop down to select one option)

Yes

No

**(If the answer to the above question is “No”, the questionnaire ends and the information will be recorded with the end time. If the answer to the above question is “Yes”, the questionnaire proceeds to part B)**

**Part B – DRUG ALLERGY**

| **DOCUMENTATION/ ADVICE/ PRACTICE**  B1. Do you currently have any documentation (allergy card, medical note, or prescription) confirming your drug allergy?  Yes  No  B2. Do you tell your doctor about your drug allergy when seeking treatment? (drop down to select one option)  Yes  No  B3: What are the drugs that you are allergic to?  Name of the first drug – until the name of 10^th^ drug (**Available documentation to be reviewed by the interviewer to verify reported information)**  **10 separate spaces to mention the names of each drug**  B4: If you cannot remember the drugs/documents not available, please mention for which conditions they were used- (drop down to select multiple options)  during an infection  for fever  for pain relief  to anaesthetize  for fits  cannot remember- if patient cannot report or documents not available  any other – If this is selected a space is provided to mention |
| --- |
| **If the interviewer finds that the interviewee is allergic to 2 or more drug types (e.g., antibiotics and NSAIDs), proceed to question B5. If not, go to question B6.**  B5: Please specify whether these different types of drugs were taken simultaneously or at separate times concerning the allergic reaction. (drop down to select one option)  Simultaneously  Separately  B6: Is the patient allergic to an antibiotic? (drop down to select one option)  Yes  No  **If the answer is “yes” to above, the questionnaire will proceed to part C. If the answer is “no” the questionnaire ends and the information will be recorded with the end time.** |
| **Part C – ANTIBIOTIC ALLERGY**  **If the interviewee is allergic to multiple antibiotics, please fill questions C1 to C7 separately for each antibiotic** |
| **ANTIBIOTIC 1**  C1:Mention the name of the antibiotic 1 to which the patient had a reaction – (drop down to select one option from the names of 37 antibiotics belonging to beta-lactams, non beta-lactams, anti-TB etc, unknown, other ( space to mention the name) |
| C2: What were the symptoms that you experienced at the time of allergic reaction to above antibiotic? (Drop down to select multiple answers)  Itching  Urticaria  Benign non-urticarial rash  Angioedema  Respiratory compromise (SOB, wheezing, chest tightness, stridor)  Faintness/ dizziness  LOC  Anaphylaxis  Serious non-immediate features (SJS/ TEN/ DRESS syndrome/AGEP/erythema multiforme/ deep organ involvement)  Isolated symptoms not suggestive of allergy (e.g. nausea, vomiting, abdominal discomfort , diarrhoea, headache)  No symptoms and family history only  Remote unknown reaction/ cannot remember  C3: What was the time of onset of the allergic reaction after taking the drug? (drop down to select one option)  <1 hour of 1^st^ dose  1-6 hours of 1^st^ dose  2^nd^ dose or later during the course of the treatment  After stopping the treatment  Indeterminate |
| C4: Risk stratification by the local PI. Risk stratification based on criteria to be confirmed by site PI- (drop down to select one option)  Low risk  High risk |
| C5: How was the antibiotic allergy diagnosed? (drop down to select one option)  Confirmed by doctor  Self-reported  **If it is confirmed by doctor, proceed to C6**  C6. How was it confirmed by doctor? (drop down to select one option)  Confirmed by oral challenge  Confirmed by skin test  Confirmed by blood test  Diagnosed based on symptoms |
| **C7 Are you allergic to more than one antibiotic?**  Yes  No  **If “Yes” the questionnaire will proceed to C1 for information regarding the additional antibiotics. (Questions will be repeated from C1-C6 for each different antibiotic)** |
| **End of questionnaire. The information is recorded with the end time.** |

**Table S2: Age, sex prevalence of reported drug allergy**

| **Age categories** | **Males (183)** | | **Females (544)** | | **Total (727)** | |
| --- | --- | --- | --- | --- | --- | --- |
|  | N | % | N | % | N | % |
| 18-<40 | 25 | 13·7 | 82 | 15·1 | 107 | 14·7 |
| 40-<50 | 22 | 12·0 | 87 | 16·0 | 109 | 15·0 |
| 50-<60 | 39 | 21·3 | 159 | 29·2 | 198 | 27·2 |
| 60-93 | 97 | 53·0 | 216 | 39·7 | 313 | 43·1 |
| Mean age (SD) | 56·9 (15·5) | | 54·5 (13·9) | | 55·1 (14·4) | |

**Table S3: District-wise prevalence of reported drug allergy in Sri Lanka**

| **District** | **Total number of participants (n)** | **Allergy to any drug (n)** | **Percentage** |
| --- | --- | --- | --- |
| Gampaha | 2625 | 203 | 7·7 |
| Colombo | 2550 | 197 | 7·7 |
| Galle | 2054 | 97 | 4·7 |
| Kandy | 2133 | 82 | 3·8 |
| Anuradhapura | 1570 | 79 | 5·0 |
| Batticaloa | 1559 | 69 | 4·4 |

**Table S4: Cross-tabulation by districts**

| Variable | Colombo | Gampaha | Galle | Anuradhapura | Batticaloa | Kandy | p-value |
| --- | --- | --- | --- | --- | --- | --- | --- |
| n | 2550 | 2625 | 2054 | 1570 | 1559 | 2133 |  |
| Allergy | 197 (7.7) | 203 (7.7) | 97 (4.7) | 79 (5.0) | 69 (4.4) | 82 (3.8) | <0.001 |
| Age |  |  |  |  |  |  | <0.001 |
| [18,40) | 585 (22.9) | 280 (10.7) | 419 (20.4) | 459 (29.2) | 550 (35.3) | 582 (27.3) |  |
| [40,50) | 425 (16.7) | 330 (12.6) | 445 (21.7) | 364 (23.2) | 343 (22.0) | 414 (19.4) |  |
| [50,60) | 562 (22.0) | 615 (23.4) | 533 (25.9) | 354 (22.5) | 317 (20.3) | 408 (19.1) |  |
| [60,93] | 978 (38.4) | 1400 (53.3) | 657 (32.0) | 393 (25.0) | 349 (22.4) | 729 (34.2) |  |
| Sex (Male) | 819 (32.1) | 1143 (43.5) | 532 (25.9) | 445 (28.3) | 401 (25.7) | 741 (34.7) | <0.001 |
| Ethnicity |  |  |  |  |  |  | <0.001 |
| Sinhalese | 2210 (86.7) | 2393 (91.2) | 1844 (89.8) | 1513 (96.4) | 47 (3.0) | 1479 (69.3) |  |
| Tamil | 234 (9.2) | 140 (5.3) | 98 (4.8) | 7 (0.4) | 1203 (77.2) | 477 (22.4) |  |
| Muslim | 104 (4.1) | 60 (2.3) | 105 (5.1) | 49 (3.1) | 307 (19.7) | 170 (8.0) |  |
| Other | 2 (0.1) | 32 (1.2) | 7 (0.3) | 1 (0.1) | 2 (0.1) | 7 (0.3) |  |
| Endocrine Dis. | 884 (34.7) | 1003 (38.2) | 166 (8.1) | 317 (20.2) | 291 (18.7) | 428 (20.1) | <0.001 |
| Allergy Conditions | 810 (31.8) | 409 (15.6) | 401 (19.5) | 465 (29.6) | 454 (29.1) | 231 (10.8) | <0.001 |
| CVD | 222 (8.7) | 496 (18.9) | 280 (13.6) | 83 (5.3) | 71 (4.6) | 162 (7.6) | <0.001 |
| Renal Condition | 40 (1.6) | 419 (16.0) | 95 (4.6) | 46 (2.9) | 18 (1.2) | 30 (1.4) | <0.001 |
| Rheumatological | 305 (12.0) | 133 (5.1) | 40 (1.9) | 54 (3.4) | 41 (2.6) | 61 (2.9) | <0.001 |
| Neurological | 152 (6.0) | 153 (5.8) | 55 (2.7) | 55 (3.5) | 17 (1.1) | 44 (2.1) | <0.001 |
| CA (Yes) | 24 (0.9) | 111 (4.2) | 39 (1.9) | 13 (0.8) | 6 (0.4) | 27 (1.3) | <0.001 |
| Chronic Urticaria | 51 (2.0) | 51 (1.9) | 57 (2.8) | 44 (2.8) | 1 (0.1) | 16 (0.8) | <0.001 |
| Chronic Liver Dis. | 17 (0.7) | 85 (3.2) | 46 (2.2) | 11 (0.7) | 2 (0.1) | 8 (0.4) | <0.001 |
| Mental illness | 20 (0.8) | 48 (1.8) | 21 (1.0) | 17 (1.1) | 9 (0.6) | 33 (1.5) | 0.001 |
| Respiratory Dis. | 3 (0.1) | 5 (0.2) | 21 (1.0) | 1 (0.1) | 0 (0.0) | 4 (0.2) | <0.001 |
| Dermatological Dis. | 18 (0.7) | 19 (0.7) | 0 (0.0) | 2 (0.1) | 4 (0.3) | 1 (0.0) | <0.001 |
| Haematological Dis. | 5 (0.2) | 15 (0.6) | 0 (0.0) | 6 (0.4) | 1 (0.1) | 3 (0.1) | 0.001 |
| Gastrointestinal Dis. | 1 (0.0) | 6 (0.2) | 0 (0.0) | 0 (0.0) | 8 (0.5) | 0 (0.0) | <0.001 |
| Hypertension | 917 (36.0) | 1189 (45.3) | 361 (17.6) | 345 (22.0) | 321 (20.6) | 556 (26.1) | <0.001 |
| Metabolic Condition | 939 (36.8) | 945 (36.0) | 137 (6.7) | 323 (20.6) | 268 (17.2) | 415 (19.5) | <0.001 |
| Other Health Cond. | 218 (8.5) | 80 (3.0) | 45 (2.2) | 9 (0.6) | 20 (1.3) | 25 (1.2) | <0.001 |
| Table 2. Frequency (n, %) of demographic and health-related characteristics of patients across districts, for affirmative ('Yes') responses. | | | | | | | |

**Table S5: Variance inflation factor**

|  | **GVIF** | **Df** | **Adjusted GVIF** |
| --- | --- | --- | --- |
| Age | 1·26 | 3 | 1·04 |
| Sex | 1·05 | 1 | 1·03 |
| Ethnicity | 1·04 | 3 | 1·01 |
| Endocrine | 1·13 | 1 | 1·06 |
| Allergic diseases | 1·01 | 1 | 1·00 |
| Cardiovascular | 1·08 | 1 | 1·04 |
| Rheumatological | 1·04 | 1 | 1·02 |
| Neurological | 1·01 | 1 | 1·00 |
| Malignant | 1·01 | 1 | 1·01 |
| Haematological | 1·00 | 1 | 1·00 |

All predictors demonstrated low collinearity (adjusted GVIF values < 2)
